# Supplementary material for: Probiotic-Induced Modulation of Microbiota Composition and Antibiotic Resistance Genes Load, an In Vitro Assessment
Source: Int J Mol Sci. 2024 Jan 13;25(2):1003. doi: 10.3390/ijms25021003 (PMC10816173; doi:10.3390/ijms25021003)
Supplement: Supplementary file 1 [file ijms-25-01003-s001.zip › ijms-2737918-supplementary.pdf]

Supplementary Material

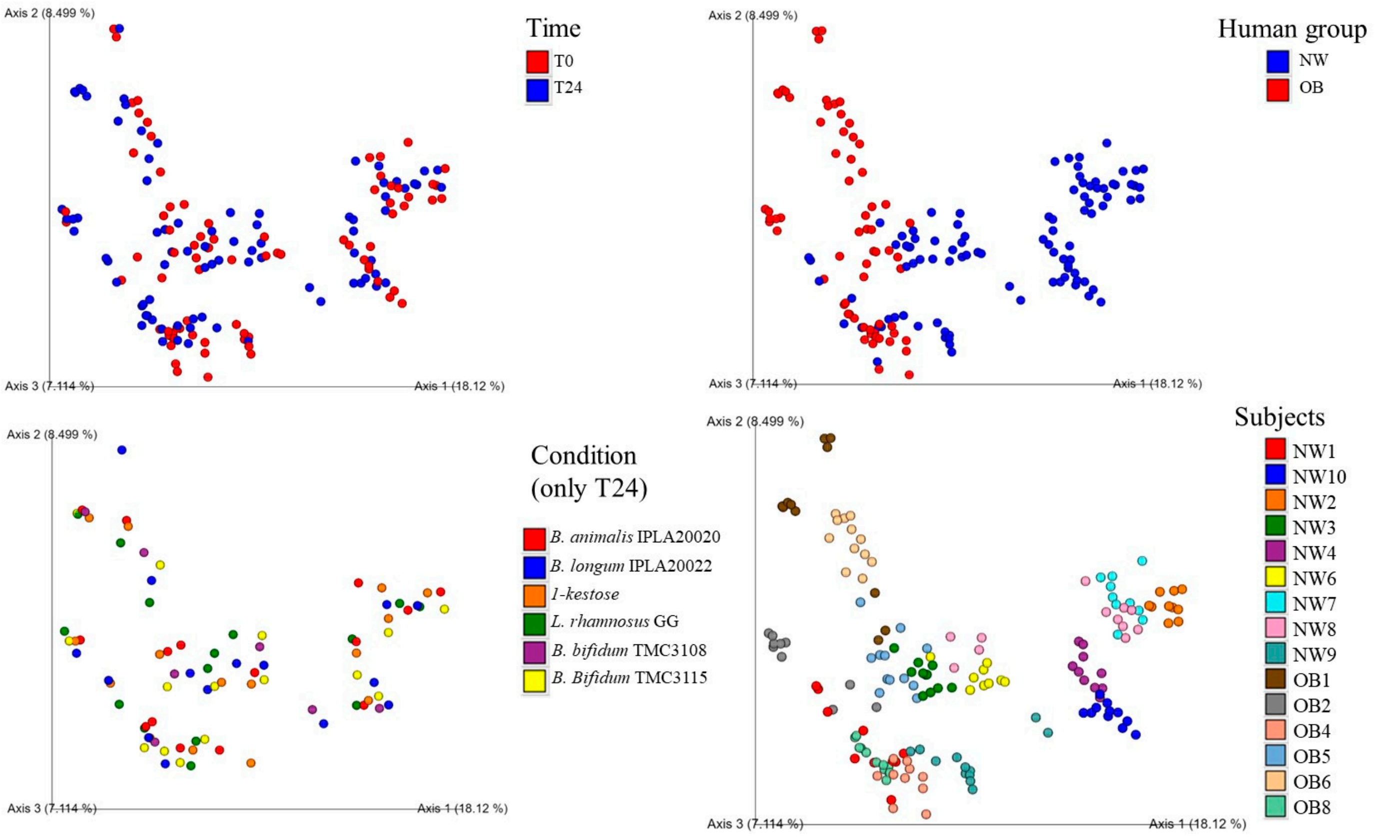

Supplementary Figure S1. The comparison of the microbiota communities through principal coordinates (PCoA) of un-weighted UniFrac distance using Emperor in QIIME 2.

Supplementary Table S1. Levels (number of copies/g) of the different antibiotic resistance genes quantified a the beginning (T=0) and end of the incubation (T=24).

	tetM			*tetO*			*blaTEM*			*blaSHV*		
	T0	T24	p-value	T0	T24	p-value	T0	T24	p-value	T0	T24	p-value
B.animalis IPLA 20020	6.19 ± 0.71	5.81 ± 0.60	0.012	5.90 ± 1.00	5.44 ± 0.97	0.008	10.06 ± 0.73	9.61 ± 0.97	0.007	6.23 ± 0.53	6.32 ± 0.65	0.109
B.longum IPLA20022	6.14 ± 0.69	5.63 ± 0.54	0.012	6.08 ± 1.01	5.43 ± 0.85	0.008	9.83 ± 0.73	9.30 ± 1.06	0.008	6.46 ± 0.74	6.23 ± 0.53	0.180
Control	5.44 ± 0.31	5.62 ± 0.39	0.401	6.16 ± 0.56	5.89 ± 0.79	0.767	9.60 ± 0.80	9.29 ± 0.73	0.028	6.49 ± 0.90	6.56 ± 0.87	0.180
L.rhamnosus GG	5.97 ± 0.61	5.49 ± 0.46	0.046	5.57 ± 0.76	5.11 ± 0.72	0.008	9.99 ± 0.64	9.59 ± 0.69	0.012	6.44 ± 0.90	6.21 ± 0.67	0.317
B.bifidum TMC3108	6.29 ± 0.41	5.14 ± 0.72	0.109	5.41 ± 1.14	5.00 ± 0.83	0.109	9.38 ± 0.92	9.19 ± 0.81	0.180	6.22 ± 0.29	6.22 ± 0.29	0.109
B.bifidum TMC3115	6.30 ± 0.48	5.72 ± 0.30	0.018	5.70 ± 0.88	4.98 ± 0.85	0.008	9.62 ± 0.88	9.08 ± 1.00	0.012	6.89 ± 1.56	6.58 ± 1.54	0.715
B.animalis IPLA 20020	5.90 ± 0.71	5.42 ± 0.83	0.028	6.27 ± 0.43	5.60 ± 0.65	0.043	9.83 ± 1.96	9.57 ± 1.98	0.028	6.67 ± 0.46	6.30 ± 0.16	0.746
B.longum IPLA20022	6.01 ± 0.74	5.45 ± 0.75	0.028	6.35 ± 0.46	5.82 ± 0.40	0.043	9.82 ± 2.05	9.31 ± 2.20	0.028	6.55 ± 0.38	6.32 ± 0.12	0.066
Control	5.83 ± 0.64	5.65 ± 0.78	0.336	6.28 ± 0.35	6.25 ± 0.33	0.249	9.89 ± 1.75	9.59 ± 1.90	0.116	6.63 ± 0.38	6.39 ± 0.00	0.223
L.rhamnosus GG	5.62 ± 0.77	5.30 ± 0.77	0.043	6.20 ± 0.33	5.64 ± 0.36	0.027	9.73 ± 2.11	9.31 ± 2.05	0.028	6.38 ± 0.03	6.33 ± 0.14	0.042
B.bifidum TMC3108	5.93 ± 0.44	4.91 ± 0.28	0.109	6.15 ± 0.18	5.20 ± 0.97	0.180	7.57 ± 0.92	6.97 ± 1.27	0.109	7.18 ± 0.35	6.13 ± 0.23	0.317
B.bifidum TMC3115	5.61 ± 0.62	5.03 ± 0.62	0.028	6.13 ± 0.36	5.62 ± 0.33	0.043	9.59 ± 1.78	9.21 ± 1.80	0.028	6.62 ± 0.40	6.58 ± 0.44	0.039

Supplementary Table S2. Results from stepwise regression analysis between significant correlated associations of family relative abundances and ARG levels performed individually for OB and NW-fecal cultures.

Group	ARG	Model	Variables	R2	β	*p*-value
OB	*tetM*	1	Bifidobacteriaceae	0.321	-0.57	0.00
		2	Bifidobacteriaceae	0.552	-0.55	0.00
			Individual		0.48	0.00
	tetO	1	Erysipelotrichaceae	0.575	-0.759	0.00
		2	Erysipelotrichaceae	0.62	-0.741	0.00
			Individual		-0.213	0.00
	blaTEM	1	Clostridiaceae 1	0.176	0.419	0.01
		2	Clostridiaceae 1	0.274	0.54	0.00
			Christensenellaceae		0.316	0.03
		3	Clostridiaceae 1	0.372	0.211	0.20
			Christensenellaceae		0.533	0.00
			Erysipelotrichaceae		-0.46	0.02
		4	Christensenellaceae	0.345	0.59	0.00
			Erysipelotrichaceae		-0.61	0.00
		5	Christensenellaceae	0.413	0.79	0.00
			Erysipelotrichaceae		-0.69	0.00
			Individual		-0.31	0.04
NW	*tetM*	1	Bifidobacteriaceae	0.554	-0.28	0.04
			Prevotellaceae		0.14	0.28
			Streptococcaceae		0.00	1.00
			Christensenellaceae		0.07	0.68
			Ruminococcaceae		0.28	0.06
			Acidaminococcaceae		0.40	0.01
			Veillonellaceae		0.00	0.99
			Enterobacteriaceae		0.08	0.74
	tetO	1	Bifidobacteriaceae	0.3999	-0.23	0.13
			Prevotellaceae		-0.03	0.83
			Ruminococcaceae		0.48	0.14
			Veillonellaceae		-0.19	0.35
			Enterobacteriaceae		0.10	0.57
		2	Bifidobacteriaceae	0.378	-0.33	0.02
			Prevotellaceae		0.09	0.43
			Ruminococcaceae		-0.09	0.45
			Enterobacteriaceae		0.44	0.00

blaTEM	1	Bifidobacteriaceae	0.521	-0.22	0.11
		Prevotellaceae		0.25	0.07
		Streptococcaceae		-0.23	0.43
		Christensenellaceae		0.50	0.01
		Ruminococcaceae		-0.20	0.19
		Acidaminococcaceae		0.37	0.03
		Veillonellaceae		0.44	0.02
		Enterobacteriaceae		-0.18	0.46
	2	Bifidobacteriaceae	0.516	-0.20	0.14
		Prevotellaceae		0.21	0.09
		Streptococcaceae		-0.07	0.74
		Christensenellaceae		0.42	0.01
		Ruminococcaceae		-0.17	0.25
		Acidaminococcaceae		0.44	0.00
		Veillonellaceae		0.42	0.02
	3	Bifidobacteriaceae	0.719	-0.18	0.07
		Prevotellaceae		0.14	0.15
		Streptococcaceae		0.12	0.43
		Christensenellaceae		0.31	0.01
		Ruminococcaceae		-0.08	0.47
		Acidaminococcaceae		0.39	0.00
		Veillonellaceae		0.04	0.78
		Lachnospiraceae		-0.51	0.00
	4	Bifidobacteriaceae	0.785	-0.19	0.04
		Prevotellaceae		0.18	0.04
		Streptococcaceae		-0.17	0.28
		Christensenellaceae		0.15	0.18
		Ruminococcaceae		-0.29	0.01
		Acidaminococcaceae		0.42	0.00
		Veillonellaceae		0.01	0.93
		Lachnospiraceae		-0.39	0.00
		Burkholderiaceae		-0.38	0.00
	5	Bifidobacteriaceae	0.777	-0.21	0.02
		Prevotellaceae		0.17	0.05
		Streptococcaceae		-0.20	0.18
		Ruminococcaceae		-0.34	0.00
		Acidaminococcaceae		0.48	0.00

		Veillonellaceae		-0.03	0.83
		Lachnospiraceae		-0.39	0.00
		Burkholderiaceae		-0.43	0.00
	6	Bifidobacteriaceae	0.809	-0.17	0.05
		Prevotellaceae		0.15	0.07
		Streptococcaceae		-0.30	0.05
		Ruminococcaceae		-0.26	0.02
		Acidaminococcaceae		0.34	0.00
		Veillonellaceae		-0.11	0.39
		Lachnospiraceae		-0.29	0.00
		Burkholderiaceae		-0.37	0.00
		Individual		-0.31	0.01
blaSHV	1	Bifidobacteriaceae	0.322	0.43	0.01
		Prevotellaceae		-0.11	0.48
		Streptococcaceae		0.23	0.52
		Christensenellaceae		0.65	0.00
		Ruminococcaceae		0.36	0.05
		Acidaminococcaceae		-0.06	0.75
		Veillonellaceae		0.30	0.16
		Enterobacteriaceae		0.14	0.63
	2	Bifidobacteriaceae	0.316	0.41	0.01
		Prevotellaceae		-0.05	0.68
		Christensenellaceae		0.71	0.00
		Ruminococcaceae		0.29	0.05
		Acidaminococcaceae		-0.11	0.54
		Veillonellaceae		0.36	0.05
		Enterobacteriaceae		0.00	0.99

β, standarized regression coefficient; R^2, adjusted coefficient of multiple determination; *p*; *p* value.
